# PSA nadir as a predictive factor for biochemical disease-free survival and overall survival following whole-gland salvage HIFU following radiotherapy failure

**DOI:** 10.1038/pcan.2016.23

**Published:** 2016-07-19

**Authors:** T T Shah, M Peters, A Kanthabalan, N McCartan, Y Fatola, J van der Voort van Zyp, M van Vulpen, A Freeman, C M Moore, M Arya, M Emberton, H U Ahmed

**Affiliations:** 1 Division of Surgery and Interventional Science, UCL, London, UK; 2 Department of Urology, Whittington Hospital NHS Trust, London, UK; 3 Department of Radiation Oncology, University Medical Centre Utrecht, Utrecht, The Netherlands; 4 Department of Urology, UCLH NHS Foundation Trust, London, UK; 5 Department of Histopathology, UCLH NHS Foundation Trust, London, UK; 6 NIHR UCLH/UCL Comprehensive Biomedical Research Centre, London, UK

## Abstract

**Background:**

Treatment options for radio-recurrent prostate cancer are either androgen-deprivation therapy or salvage prostatectomy. Whole-gland high-intensity focussed ultrasound (HIFU) might have a role in this setting.

**Methods:**

An independent HIFU registry collated consecutive cases of HIFU. Between 2005 and 2012, we identified 50 men who underwent whole-gland HIFU following histological confirmation of localised disease following prior external beam radiotherapy (2005–2012). No upper threshold was applied for risk category, PSA or Gleason grade either at presentation or at the time of failure. Progression was defined as a composite with biochemical failure (Phoenix criteria (PSA>nadir+2 ng ml^−1^)), start of systemic therapies or metastases.

**Results:**

Median age (interquartile range (IQR)), pretreatment PSA (IQR) and Gleason score (range) were 68 years (64–72), 5.9 ng ml^−1^ (2.2–11.3) and 7 (6–9), respectively. Median follow-up was 64 months (49–84). In all, 24/50 (48%) avoided androgen-deprivation therapies. Also, a total of 28/50 (56%) achieved a PSA nadir <0.5 ng ml^−1^, 15/50 (30%) had a nadir ⩾0.5 ng ml^−1^ and 7/50 (14%) did not nadir (PSA non-responders). Actuarial 1, 3 and 5-year progression-free survival (PFS) was 72, 40 and 31%, respectively. Actuarial 1, 3 and 5-year overall survival (OS) was 100, 94 and 87%, respectively. When comparing patients with PSA nadir <0.5 ng ml^−1^, nadir ⩾0.5 and non-responders, a statistically significant difference in PFS was seen (*P*<0.0001). Three-year PFS in each group was 57, 20 and 0%, respectively. Five-year OS was 96, 100 and 38%, respectively. Early in the learning curve, between 2005 and 2007, 3/50 (6%) developed a fistula. Intervention for bladder outlet obstruction was needed in 27/50 (54%). Patient-reported outcome measure questionnaires showed incontinence (any pad-use) as 8/26 (31%).

**Conclusions:**

In our series of high-risk patients, in whom 30–50% may have micro-metastases, disease control rates were promising in PSA responders, however, with significant morbidity. Additionally, post-HIFU PSA nadir appears to be an important predictor for both progression and survival. Further research on focal salvage ablation in order to reduce toxicity while retaining disease control rates is required.

## Introduction

In the United Kingdom, every year up to one-quarter of men diagnosed with prostate cancer undergo external beam radiotherapy (EBRT).^1^ EBRT is an effective radical form of therapy; however, similar to other therapies it can sometimes fail. Approximately 1 in 4 to 1 in 3 develop biochemical failure. Of these, it is estimated that up to 50% may have localised recurrence, which could be suitable for local salvage treatment.^2, 3, 4^ However, the majority receive androgen-deprivation therapy (ADT) alone,^3^ which can confer systemic harms as well as cost, especially when castrate resistance occurs after a median of approximately 2–3 years.^5, 6^

Local salvage treatment options include salvage radical prostatectomy (SRP), cryotherapy, brachytherapy and high-intensity focussed ultrasound (HIFU). These options could not only provide a further curative strategy but defer the commencement of ADT, which would in turn delay the onset of castrate resistance and confer a cost benefit.^7, 8, 9^ These salvage options are at various stages of evaluation. We update our single-centre experience of salvage whole-gland HIFU with medium-term follow-up and aim to evaluate predictive factors for its success or failure.

## Materials And Methods

Institutional review board exemption was granted by our local University College London Hospital research and development committee. All patients were placed in a prospectively maintained HIFU registry. Between 2005 and 2012, 50 consecutive men were identified who had undergone whole-gland HIFU following histological confirmation of localised disease after prior EBRT. All underwent bone scan, pelvic/prostate multiparametric magnetic resonance imaging (MRI) and cross-sectional computed tomography (with or without choline or fluorodeoxyglucose positron emission tomography) to rule out metastases. No upper threshold was applied for risk category, PSA or Gleason grade either at presentation or at time of failure. Limited data were available on pre-EBRT characteristics including the radiotherapy dose despite approaching referring centres and was thus not included in the statistical analyses.

All men underwent a standardised HIFU protocol:^10^ Patients were placed in the lithotomy position with a Sonablate 500 device (Sonacare, Charlotte, NC, USA) with a rectally placed treatment probe. Treatment planning took place using the proprietary software and once started the software moved the transducer automatically to allow complete coverage of the prostate. Real-time ultrasound scanning was used to visualise the prostate during treatment and power was adjusted according to changes seen. Acoustic pulses measuring 3 × 3 × 10 mm^3^ with slight overlap moved sequentially through the prostate with 3 s ‘on' time exposures and 6 s ‘off' time exposures. The prostate was divided into six blocks, left and right with corresponding anterior, middle and posterior. A 4-cm focal length probe was used for anterior and middle block treatment and a 3-cm probe for posterior block treatment.

All patients had a suprapubic catheter placed postoperatively, with a planned removal 2–6 weeks after treatment dependent on individual patient urethral voiding function.

All were given aminoglycoside and cephalosporin antibiotics at anesthetic induction and quinolone antibiotics for 7 days postoperatively.

Follow-up occurred with 3-monthly clinic visits in the first year followed by 6-monthly thereafter. Up to two salvage HIFU re-treatments were permitted as part of the salvage strategy. PSA tests were performed at these visits and patients were asked to complete patient-reported outcome measures (PROM) questionnaires. Routine biopsies were offered; however, no patient with a stable PSA opted for these and thus biopsies were performed only if there was a rising PSA. In addition, patients with a rising PSA were offered a multiparametric MRI prior to the biopsy.

Progression was defined as a composite outcome of biochemical recurrence using the Phoenix definition (PSA>nadir+2 ng ml^−1^) or start of ADT/second-line systemic treatments or development of metastases or cancer-specific mortality.

### Statistical analysis

Variables with a normal distribution are presented as mean (±s.d.), skewed distributed variables as medians with interquartile ranges (IQR) and categorical variables as absolute numbers with percentages. Kaplan–Meier analysis was performed to assess the freedom from several outcomes: biochemical failure, development of metastases, initiation of ADT, or a combination of these three outcomes as a composite progression end point. Statistical differences between subgroups were assessed with the log-rank test. Determinants of the composite progression end point were further analysed in univariable analysis with Cox-proportional hazards regression. Hazard ratios with 95% confidence intervals were obtained. Factors taken forward into the multivariable analysis, that is, the nadir value after salvage HIFU, were corrected for other determinants (with *P*<0.25 from univariable analysis) to assess their independent value in predicting the composite end point. Statistical significance was set at *P*⩽0.05. All analyses were performed using the R language environment (version 3.1.2) for statistical computing (using the survival, rms and survMisc packages).^11^

## Results

### Pre-EBRT demographics

Limited data were available on the original diagnosis. Median Pre-EBRT PSA was 17 ng ml^−1^ (IQR 11–28.5) (available in 50%) and median pre-EBRT Gleason score was 6 (range 4–8) (available in 80%).

The treatment year for EBRT was available in 55% and this ranged from 1996 to 2005 with the median year being 2002, while radiotherapy dosing was available in only 27% and ranged from 50 Gy in 20 fractions to 72.5 Gy in 35 fractions with a median value of 57.5 Gy.

Based on this information, the median disease-free survival interval calculated from the date of original EBRT to the date of salvage HIFU was 80 months (IQR 55–102).

### Baseline pre-salvage HIFU demographics

Median age (IQR), median pretreatment PSA (IQR) and median Gleason score (range) were 68 years (IQR 64–72), 5.9 ng ml^−1^ (IQR 2.2–11.3) and 7 (82% were Gleason ⩾3+4, range 6–9), respectively. In all, 33/50 (66%) had localised T1c–T2c disease while 17/50 (24%) had radiological T3a/b disease. Median follow-up was 64 months (IQR 49–84) (Table 1). Twenty (40%) underwent biopsies. These were all non-protocol and were performed for either a rising PSA or high posttreatment nadir. Also, 12/20 (70%) were positive for significant cancer (Gleason score ⩾7), 2/14 (%) were positive with insignificant cancer (Gleason score=6) and 6/20 (30%) were negative.

Re-treatment occurred in 7/50 (14%) once and 2/50 (4%) had two re-treatments. Six of these men had positive postsalvage HIFU biopsies showing significant cancer while 2 only had a rising PSA and a multiparametric MRI showing residual disease but refused confirmatory biopsies prior to re-treatment. Their disease characteristics on MRI matched the characteristics of the original tumour and it was deemed to be residual cancer. The positive predictive value for MRI also appears to be high in the post-HIFU setting.^12, 13, 14, 15, 16^

### Primary outcomes

Overall, 35/50 (70%) experienced biochemical failure, 26/50 (52%) were started on ADT, 12/50 (24%) developed metastases and 9/50 (18%) died (cause of death was not available). Overall, composite progression occurred in 38/50 (76%).

**Figure 1:**
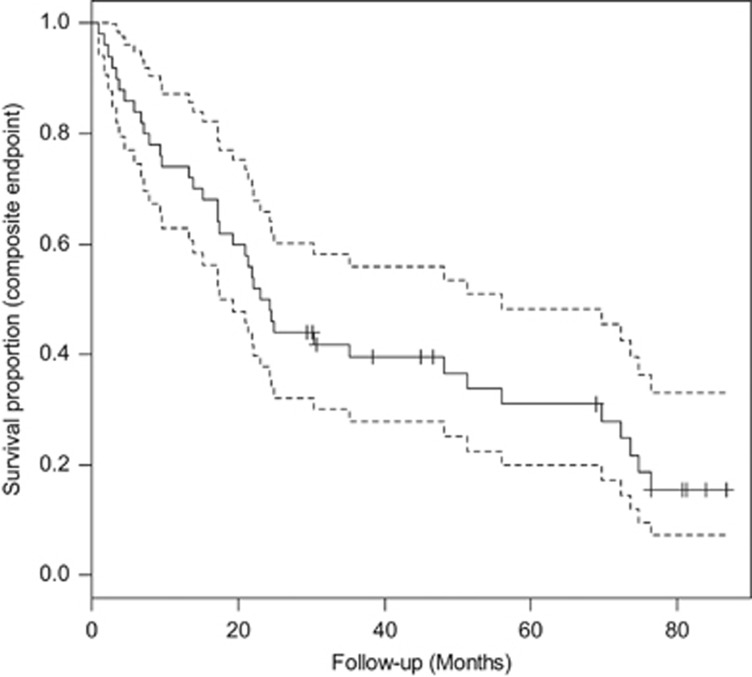
Progression-free survival for the whole cohort of 50 patients.

**Figure 2:**
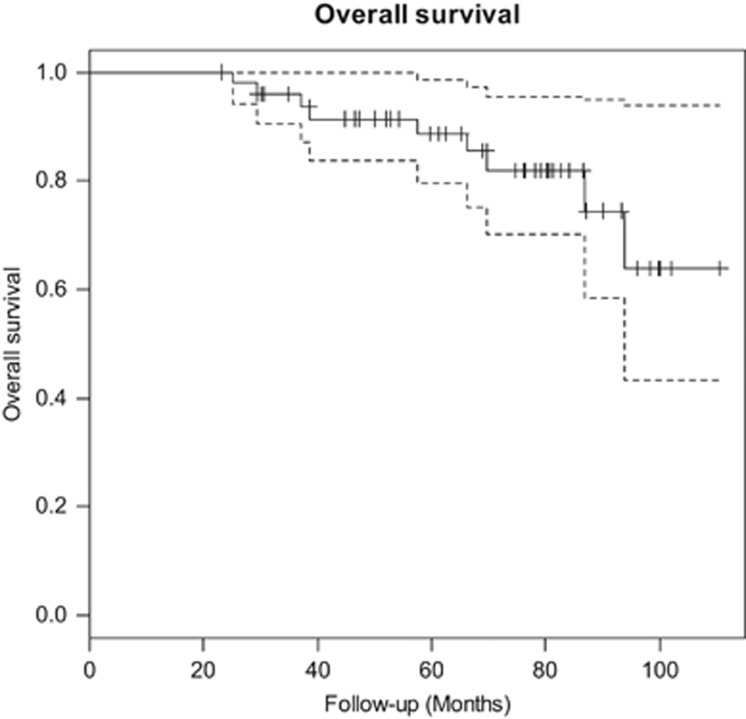
Overall survival for the whole cohort of 50 patients.

In all, 28/50 (56%) patients achieved a PSA nadir of <0.5 ng ml^−1^, 15/50 (30%) had a nadir ⩾0.5 ng ml^−1^ and 7/50 (14%) did not achieve a nadir (PSA non-responders). Actuarial 1, 3 and 5 progression-free survival (PFS) was 72, 40 and 31%, respectively (Figure 1). Analysis with PSA non-responders removed resulted in 1-, 3- and 5-year actuarial PFS of 86, 47 and 37%, respectively. Actuarial 1-, 3- and 5-year overall survival (OS) was 100, 94 and 87%, respectively (Figure 2).

### Predictive factors

**Figure 3:**
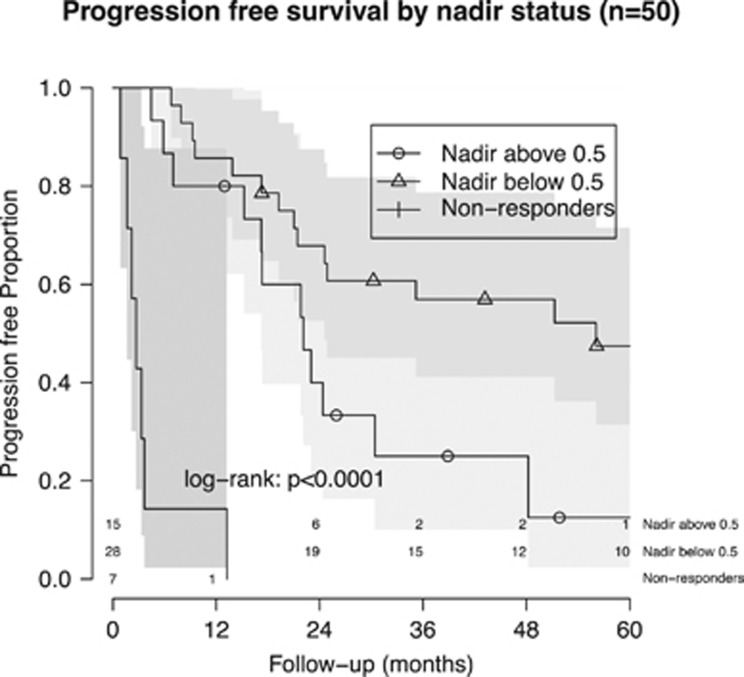
Progression-free survival by nadir status.

**Figure 4:**
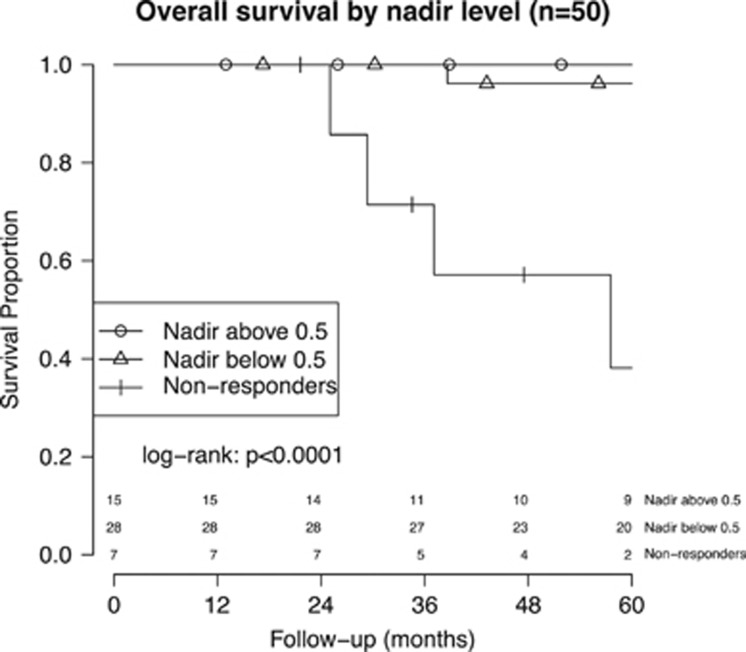
Overall survival by nadir status.

Univariable and multivariable Cox-regression analysis revealed that the only significant variable for either PFS or OS was postoperative PSA nadir (Table 2). When comparing patients with PSA nadir <0.5 ng ml^−1^, nadir ⩾0.5 and non-responders, a statistically significant difference in PFS was seen (*P*<0.0001). Three-year PFS in each group was 57, 20 and 0%, respectively (Figure 3). Five-year OS was 96, 100 and 38%, respectively (Figure 4).

### Secondary outcomes

#### MRI outcomes

A total of 36/50 patients underwent a postsalvage HIFU MRI to assess for any residual disease. In all, 10/36 (28%) had a negative scan, 4/10 underwent confirmatory biopsy and 2/4 (50%) were positive for significant cancer. Also, 20/36 (56%) had a positive scan and confirmatory biopsy was positive in all 9/9 patients who underwent it. The remaining 6/36 (17%) had either an equivocal result or images were not suitable for analysis; 2/6 underwent biopsy and these were negative in both.

#### Functional outcomes

Twenty-nine patients completed PROM questionnaires. Three patients had preexisting pad usage, and thus with their data removed, patient-reported urinary incontinence (any pad-use) was 8/26 (31%). Symptoms of bladder outlet obstruction were common and intervention in the form of a bladder neck incision, transurethral resection or urethral dilatation was needed in 27/50 patients (54%). Reviewing their results showed that they were no more likely to report incontinence than those who did not undergo intervention.

When assessing International Index of Erectile Function (IIEF)-15, although 29 patients returned at least one questionnaire only 13 patients returned both one preoperative and one postoperative questionnaire. Reviewing only the results for these patients demonstrated that the majority had preexisting severe erectile dysfunction with a pretreatment median IIEF-15 score of 9 (mean=15.3). Over a 12-month follow-up period, there was a non-significant median 3-point (mean 7 point) drop in IIEF-15 score.

Early in the learning curve, between 2005 and 2007, 2/41 developed a recto-urethral fistula after one salvage HIFU; a further 1/9 developed a fistula after a redo-HIFU. Overall, 3/50 (6%) developed a fistula. Two were managed with a diversion or closure along with an SRP while one was managed conservatively. In addition, 3/50 (6%) developed osteonecrosis of the pubic symphysis needing prolonged antibiotic treatment.

## Discussion

### Summary

Our results demonstrate a relatively low 5-year PFS of 31%, although OS was high with 87% of men still alive after 5 years. This decreased to 64% at 8 years but is comparable to the survival outcomes of patients undergoing SRP.^17^ Our results also highlight the discrepancy between disease progression, largely defined by biochemical failure, and survival. It must be noted that we had very few selection criteria for patients undergoing salvage HIFU. In patients who obtained a nadir of <0.5 ng ml^−1^, 3-year PFS was 57% compared with 20% in those with a nadir >0.5 ng ml^−1^ or 0% in non-responders. Side effects within our series were also high as with other salvage therapies in this high-risk group.

### Limitations

First, our sample size was relatively small, hence reducing the power of the statistical and multivariable analyses. Also owing to lack of data from initial diagnosis including radiotherapy dose, we were unable to use these parameters in our analysis. Second, it is possible that the true incontinence and erectile dysfunction rates may differ as we had incomplete PROM data in 42% of patients. However, our figures appear comparable to results from larger studies such as by Murat *et al.*^18^ and Crouzet *et al.*^19^ and the use of validated patient self-reported questionnaires does add a degree of validity to our results. Third, there is no PSA criteria that has been validated in this salvage setting to define biochemical failure. In our study, we used the Phoenix criteria as the majority of patients had failed EBRT based on this criterion that is the most commonly used definition in studies assessing minimally invasive salvage therapies. In addition, we used a more expansive composite outcome to define failure, which included not only clinical progression, that is, metastases/death but also clinician-determined need for initiation of second-line treatments, regardless of PSA failure, such as ADT. Thus our PFS outcomes may seem higher than studies that have only reported biochemical disease-free survival. Fourth, not all patients underwent confirmatory post-HIFU biopsy. In an ideal study, all patients would undergo posttreatment biopsies. Although we offered all patients biopsy, our data mirrors practice where patients with a stable PSA tend to refuse biopsy and even a proportion of patients with clinical suspicion for recurrence choose not to undergo biopsy and rather opt for continued PSA surveillance or proceed directly to next line treatment, that is, hormones. Furthermore, some patients developed metastases on re-staging scans and thus biopsies in these patients was deemed unnecessary. Finally, cause of death was not available, although it is likely that many of these were prostate cancer related as 5/9 had developed metastases while 3/9 patients had biochemical failure only and 1/9 had no evidence of progression.

### Clinical implications

Treatment options for patients with radio-recurrent disease are limited and historically have consisted of observation, ADT or SRP. SRP is a potentially curative procedure but is technically challenging owing to radiation-induced fibrosis and obliteration of tissue planes. Oncological outcomes range from a biochemical PFS of 47–82% at 5 years and OS of 54–89% at 10 years. However, complications are common with a 0–43% transfusion rate, erectile dysfunction in 80–100%, incontinence in 21–90%, anastomotic stricture in 7–41% and rectal injury in 0–28%.^17, 20^

Minimally invasive therapies are currently under evaluation, including brachytherapy, cryotherapy and HIFU. Our results are in keeping with previously published larger whole-gland salvage HIFU series by Murat *et al.*^18^ and Crouzet *et al.*^19^ consisting of 167 and 290 patients, respectively. Murat *et al.*^18^ initially reported a 3-year PFS of 53, 42 and 25% for (D'Amico) low, intermediate and high-risk patients, respectively, with a 5-year OS of 84%. Subsequently, Crouzet *et al.*^19^ reported 5-year PFS rates of 45, 31 and 21% for D'Amico low-, intermediate- and high-risk prostate cancer, respectively, and a 7-year cancer-specific survival of 79.6%.

However, unlike these studies we did not find, after univariable and multivariable analysis, that any pretreatment variable were predictors of either progression or survival. In our series, only postoperative PSA nadir was seen to be a strong predictor and patients with a nadir >0.5 ng ml^−1^ (or PSA non-responders) had a poorer prognosis when compared with patients with PSA nadir <0.5 ng ml^−1^. These findings could be due to the smaller sample size in our series or they may represent the difficulty in accurate risk stratification after radiotherapy and the inability of current imaging modalities to detect micrometastases that have a high prevalence in this group of men.^21, 22^ We estimate that 30–50% of men may harbour micrometastases at presentation with recurrence. This is based on the fact that, even in the presence of local treatment such as salvage prostatectomy, 10-year biochemical and metastases-free survival can be as low as 28% and 77%, respectively.^17, 23^

Unfortunately, similar to SRP, the poor side effect profile for whole-gland salvage HIFU has limited its use and recent work has focussed on focal therapy of the recurrence. The aim of a tissue-preserving strategy is to achieve similar oncological outcomes by treating only the area of recurrence but with a significantly improved side effect profile. This would be in keeping with the evidence that the site of recurrence is usually related to the location of the original tumour or ‘index' lesion.^24^ Our group has previously reported on 39 patients undergoing focal salvage HIFU. The early oncological outcomes seem comparable with a 1- and 2-year PFS of 69% and 49%, respectively. Side effects also appear to be lower than whole-gland salvage HIFU: 87% were pad-free, 23% needed intervention for bladder outflow obstruction, and 2.6% had a fistula after one salvage focal HIFU and 0% after a redo-HIFU.^25^ In addition, there is encouraging data from the focal salvage cryotherapy registry showing 1-, 3- and 5-year baseline disease-free survival of 95.3, 72.4 and 46.5%, respectively, with associated low rates of incontinence (5.5%), erectile function (50%) and fistulae (3.3%).^26^

It is evident that further research is required. Although comparative effectiveness trials are ideal, a recent randomised controlled trial of whole-gland salvage cryotherapy versus watchful waiting±ADT failed to accrue.^27^ We have since started recruiting into a study (FORECAST, NCT01883128, UKCRN15936) that aims to prospectively assess 177 patients for radiorecurrent prostate cancer initially with bone scintigraphy, positron emission tomography–computed tomography, whole-body MRI and template mapping biopsies with subsequent salvage focal ablation with HIFU or cryotherapy in those with suitable parameters.^28^ In addition to the side effect profile, another benefit of focal ablation may be an anticancer immune response, and within FORECAST, patients with oligometastatic disease will be offered ‘cytoreductive' focal therapy with local salvage treatment in addition to ADT.^29^

With reference to the learning curve, Rébillard *et al.*^30^ in 2003 commented on a learning curve of 10–15 cases. We are not aware of any more recent evidence in the literature regarding the learning curve for prostate HIFU in its current form. Over the past 10 years, HIFU has developed from early stage 1 toxicity studies into large prospective stage 2b series/trials assessing efficacy. Progression through these stages has allowed development and refinement of both the technique and the equipment. We have yet to define a learning curve for this complex procedure but certainly it should be coached in expert centres who have extensive experience of primary HIFU.

## Conclusion

Whole-gland salvage HIFU carries significant morbidity. In our series of high-risk patients, in whom we estimate, based on the literature, that 30–50% may have micro-metastases at the time of presentation, disease control rates were promising in PSA responders.^17, 21, 22, 23, 31^ Furthermore, PSA nadir after treatment appears to be an important predictor for both progression and survival. Further research on focal salvage ablation as a strategy to reduce toxicity while retaining disease control rates is required.

## Figures and Tables

**Table 1:** Descriptive statistics

*Determinant*	*Mean/median/*n	*S.d./IQR/%*
Age pre-HIFU, median, (IQR)	68	64–72
ADT use, *N* (%)	31	62%
PSA pre-HIFU, median, ng ml^−1^, (IQR)	6.3	2.3–10.8
Nadir post-HIFU, median, ng ml^−1^, (IQR)	0.12	0.05–0.83
No nadir after salvage HIFU, *N* (%)	7	15%
Progression (composite), *N* (%)	38	76%
BF, *N* (%)	35	70%
Metastases, *N* (%)	12	24%
Initiation ADT, *N* (%)	26	52%
Death, *N* (%)	9	18%

Abbreviations: ADT, androgen-deprivation therapy; BF, biochemical failure; HIFU, high-intensity focussed ultrasound; IQR, interquartile range.

**Table 2:** Cox-regression analysis for composite end point/progression

*Determinant*	*Univariable (HR, 95% CI (*P))	*Multivariable (HR, 95% CI)*
Age	0.94 (0.88–0.99 (*P*=0.03))	0.96 (0.90–1.01 (*P*=0.13))
ADT	2.15 (1.06–4.37 (*P*=0.04))	1.69 (0.80–3.61 (*P*=0.17))
PSA	1.00 (0.97–1.04 (*P*=0.95))	—
		
*PSA nadir*		
>0.5 versus <0.5	2.63 (1.22–5.69 (*P*=0.01))	2.61 (1.19–5.73 (*P*=0.02))
No nadir versus <0.5	35.11 (10.64–115.81 (*P*<0.0001))	28 (8.29–94.53 (*P*<0.0001))
		
NCCN risk group (high versus intermediate)	1.22 (0.62–2.40 (*P*=0.57))	—

Abbreviations: ADT, androgen-deprivation therapy; CI, confidence interval; HR, hazard ratio; NCCN, National Comprehensive Cancer Network.

## References

[bib1] UK CR. Prostate Cancer Statistics, 2011. Cancer Research UK, http://www.cancerresearchuk.org/health-professional/cancer-statistics/statistics-by-cancer-type/prostate-cancer. Accessed February 2016.

[bib2] Pollack A, Hanlon AL, Horwitz EM, Feigenberg SJ, Uzzo RG, Hanks GE. Prostate cancer radiotherapy dose response: an update of the fox chase experience. J Urol 2004; 171: 1132–1136. 14767286 10.1097/01.ju.0000111844.95024.74

[bib3] Agarwal PK, Sadetsky N, Konety BR, Resnick MI, Carroll PR, Cancer of the Prostate Strategic Urological Research Endeavor. Treatment failure after primary and salvage therapy for prostate cancer: likelihood, patterns of care, and outcomes. Cancer 2008; 112: 307–314. 18050294 10.1002/cncr.23161

[bib4] Widmark A, Klepp O, Solberg A, Damber JE, Angelsen A, Fransson P et al. Endocrine treatment, with or without radiotherapy, in locally advanced prostate cancer (SPCG-7/SFUO-3): an open randomised phase III trial. Lancet 2009; 373: 301–308. 19091394 10.1016/S0140-6736(08)61815-2

[bib5] Sharifi N, Dahut WL, Steinberg SM, Figg WD, Tarassoff C, Arlen P et al. A retrospective study of the time to clinical endpoints for advanced prostate cancer. BJU Int 2005; 96: 985–989. 16225513 10.1111/j.1464-410X.2005.05798.x

[bib6] Ross RW, Xie W, Regan MM, Pomerantz M, Nakabayashi M, Daskivich TJ et al. Efficacy of androgen deprivation therapy (ADT) in patients with advanced prostate cancer: association between Gleason score, prostate-specific antigen level, and prior ADT exposure with duration of ADT effect. Cancer 2008; 112: 1247–1253. 18293426 10.1002/cncr.23304

[bib7] Bayoumi AM, Brown AD, Garber AM. Cost-effectiveness of androgen suppression therapies in advanced prostate cancer. J Natl Cancer Inst 2000; 92: 1731–1739. 11058616 10.1093/jnci/92.21.1731

[bib8] Boyd KA, Jones RJ, Paul J, Birrell F, Briggs AH, Leung HY. Decision analytic cost-effectiveness model to compare prostate cryotherapy to androgen deprivation therapy for treatment of radiation recurrent prostate cancer. BMJ Open 2015; 5: e007925. 10.1136/bmjopen-2015-007925 PMC4611206 26482768

[bib9] Friedlander DF, Gu X, Prasad SM, Lipsitz SR, Nguyen PL, Trinh QD et al. Population-based comparative effectiveness of salvage radical prostatectomy vs cryotherapy. Urology 2014; 83: 653–657. 24581527 10.1016/j.urology.2013.11.019

[bib10] Illing RO, Leslie TA, Kennedy JE, Calleary JG, Ogden CW, Emberton M. Visually directed high-intensity focused ultrasound for organ-confined prostate cancer: a proposed standard for the conduct of therapy. BJU Int 2006; 98: 1187–1192. 17125476 10.1111/j.1464-410X.2006.06509.x

[bib11] R-Core-TeamA Language and Environment for Statistical Computing. R Foundation for Statistical Computing: Vienna, Austria, 2015; Available from http://www.r-project.org/.

[bib12] Kirkham AP, Emberton M, Hoh IM, Illing RO, Freeman AA, Allen C. MR imaging of prostate after treatment with high-intensity focused ultrasound. Radiology 2008; 246: 833–844. 18223121 10.1148/radiol.2463062080

[bib13] Rouviere O, Girouin N, Glas L, Ben Cheikh A, Gelet A, Mege-Lechevallier F et al. Prostate cancer transrectal HIFU ablation: detection of local recurrences using T2-weighted and dynamic contrast-enhanced MRI. Eur Radiol 2010; 20: 48–55. 19690866 10.1007/s00330-009-1520-5

[bib14] Ben Cheikh A, Girouin N, Ryon-Taponnier P, Mege-Lechevallier F, Gelet A, Chapelon JY et al. MR detection of local prostate cancer recurrence after transrectal high-intensity focused US treatment: preliminary results. J Radiol 2008; 89: 571–577. 18535498 10.1016/s0221-0363(08)71483-5

[bib15] Kim CK, Park BK, Lee HM, Kim SS, Kim E. MRI techniques for prediction of local tumor progression after high-intensity focused ultrasonic ablation of prostate cancer. AJR Am J Roentgenol 2008; 190: 1180–1186. 18430829 10.2214/AJR.07.2924

[bib16] Cirillo S, Petracchini M, D'Urso L, Dellamonica P, Illing R, Regge D et al. Endorectal magnetic resonance imaging and magnetic resonance spectroscopy to monitor the prostate for residual disease or local cancer recurrence after transrectal high-intensity focused ultrasound. BJU Int 2008; 102: 452–458. 18476973 10.1111/j.1464-410X.2008.07633.x

[bib17] Chade DC, Eastham J, Graefen M, Hu JC, Karnes RJ, Klotz L et al. Cancer control and functional outcomes of salvage radical prostatectomy for radiation-recurrent prostate cancer: a systematic review of the literature. Eur Urol 2012; 61: 961–971. 22280856 10.1016/j.eururo.2012.01.022

[bib18] Murat FJ, Poissonnier L, Rabilloud M, Belot A, Bouvier R, Rouviere O et al. Mid-term results demonstrate salvage high-intensity focused ultrasound (HIFU) as an effective and acceptably morbid salvage treatment option for locally radiorecurrent prostate cancer. Eur Urol 2009; 55: 640–647. 18508188 10.1016/j.eururo.2008.04.091

[bib19] Crouzet S, Murat FJ, Pommier P, Poissonnier L, Pasticier G, Rouviere O et al. Locally recurrent prostate cancer after initial radiation therapy: early salvage high-intensity focused ultrasound improves oncologic outcomes. Radiother Oncol 2012; 105: 198–202. 23068708 10.1016/j.radonc.2012.09.014

[bib20] Kimura M, Mouraviev V, Tsivian M, Mayes JM, Satoh T, Polascik TJ. Current salvage methods for recurrent prostate cancer after failure of primary radiotherapy. BJU Int 2010; 105: 191–201. 19583717 10.1111/j.1464-410X.2009.08715.x

[bib21] Kosuri S, Akhtar NH, Smith M, Osborne JR, Tagawa ST. Review of salvage therapy for biochemically recurrent prostate cancer: the role of imaging and rationale for systemic salvage targeted anti-prostate-specific membrane antigen radioimmunotherapy. Adv Urol 2012; 2012: 921674. 22693495 10.1155/2012/921674 PMC3368159

[bib22] Morgan PB, Hanlon AL, Horwitz EM, Buyyounouski MK, Uzzo RG, Pollack A. Timing of biochemical failure and distant metastatic disease for low-, intermediate-, and high-risk prostate cancer after radiotherapy. Cancer 2007; 110: 68–80. 17520705 10.1002/cncr.22755 PMC1950742

[bib23] Chade DC, Shariat SF, Cronin AM, Savage CJ, Karnes RJ, Blute ML et al. Salvage radical prostatectomy for radiation-recurrent prostate cancer: a multi-institutional collaboration. Eur Urol 2011; 60: 205–210. 21420229 10.1016/j.eururo.2011.03.011 PMC3124574

[bib24] Jalloh M, Leapman MS, Cowan JE, Shinohara K, Greene KL, Roach M 3rd et al. Patterns of local failure following radiation therapy for prostate cancer. J Urol 2015; 194: 977–982. 25983194 10.1016/j.juro.2015.04.111

[bib25] Ahmed HU, Cathcart P, McCartan N, Kirkham A, Allen C, Freeman A et al. Focal salvage therapy for localized prostate cancer recurrence after external beam radiotherapy: a pilot study. Cancer 2012; 118: 4148–4155. 22907704 10.1002/cncr.27394

[bib26] Li YH, Elshafei A, Agarwal G, Ruckle H, Powsang J, Jones JS. Salvage focal prostate cryoablation for locally recurrent prostate cancer after radiotherapy: initial results from the cryo on-line data registry. Prostate 2015; 75: 1–7. 25283814 10.1002/pros.22881

[bib27] Salji M, Jones R, Paul J, Birrell F, Dixon-Hughes J, Hutchison C et al. Feasibility study of a randomised controlled trial to compare (deferred) androgen deprivation therapy and cryotherapy in men with localised radiation-recurrent prostate cancer. Br J Cancer 2014; 111: 424–429. 24946001 10.1038/bjc.2014.316 PMC4119985

[bib28] Kanthabalan A, Shah T, Arya M, Punwani S, Bomanji J, Haroon A et al. The FORECAST Study - Focal Recurrent Assessment and Salvage Treatment for Radiorecurrent Prostate Cancer. Contemp Clin Trials 2015. pii: S1551-7144(15)30038-0. doi: 10.1016/j.cct.2015.07.004 (e-pub ahead of print) PubMed PMID: 26184343. 10.1016/j.cct.2015.07.004 26184343

[bib29] Bastianpillai C, Petrides N, Shah T, Guillaumier S, Ahmed HU, Arya M. Harnessing the immunomodulatory effect of thermal and non-thermal ablative therapies for cancer treatment. Tumour Biol 2015; 36: 9137–9146. 26423402 10.1007/s13277-015-4126-3

[bib30] Rébillard X, Davin JL, Soulié M. Comité de Cancérologie de l'Association Française d'Urologie. [Treatment by HIFU of prostate cancer: survey of literature and treatment indications]. Prog Urol 2003; 13: 1428–1456. 15000326

[bib31] Lee WR, Hanks GE, Hanlon A. Increasing prostate-specific antigen profile following definitive radiation therapy for localized prostate cancer: clinical observations. J Clin Oncol 1997; 15: 230–238. 8996147 10.1200/JCO.1997.15.1.230

